# Interactions between physical exercise, associative memory, and genetic risk for Alzheimer’s disease

**DOI:** 10.1093/cercor/bhae205

**Published:** 2024-05-28

**Authors:** Kinga Igloi, Blanca Marin Bosch, Noémie Kuenzi, Aurélien Thomas, Estelle Lauer, Aurélien Bringard, Sophie Schwartz

**Affiliations:** Department of Fundamental Neurosciences, University of Geneva, CH-1211 Geneva, Switzerland; Swiss Center for Affective Sciences, University of Geneva, CH-1211 Geneva, Switzerland; Department of Fundamental Neurosciences, University of Geneva, CH-1211 Geneva, Switzerland; Department of Fundamental Neurosciences, University of Geneva, CH-1211 Geneva, Switzerland; Faculty Unit of Toxicology, CURML, Faculty of Biology and Medicine, University of Lausanne, CH-1015 Lausanne, Switzerland; Unit of Forensic Toxicology and Chemistry, CURML, Lausanne and Geneva University Hospitals, Lausanne, CH-1011 Geneva, Switzerland; Unit of Forensic Toxicology and Chemistry, CURML, Lausanne and Geneva University Hospitals, Lausanne, CH-1011 Geneva, Switzerland; Department of Pneumology, Geneva University Hospitals, CH-1011 Geneva, Switzerland; Department of Fundamental Neurosciences, University of Geneva, CH-1211 Geneva, Switzerland; Swiss Center for Affective Sciences, University of Geneva, CH-1211 Geneva, Switzerland; Geneva Neuroscience Center, University of Geneva, CH-1211 Geneva, Switzerland

**Keywords:** Alzheimer’s disease, endocannabinoids, fMRI, memory, physical exercise

## Abstract

The ε4 allele of the APOE gene heightens the risk of late onset Alzheimer’s disease. ε4 carriers, may exhibit cognitive and neural changes early on. Given the known memory-enhancing effects of physical exercise, particularly through hippocampal plasticity via endocannabinoid signaling, here we aimed to test whether a single session of physical exercise may benefit memory and underlying neurophysiological processes in young ε3 carriers (ε3/ε4 heterozygotes, risk group) compared with a matched control group (homozygotes for ε3). Participants underwent fMRI while learning picture sequences, followed by cycling or rest before a memory test. Blood samples measured endocannabinoid levels. At the behavioral level, the risk group exhibited poorer associative memory performance, regardless of the exercising condition. At the brain level, the risk group showed increased medial temporal lobe activity during memory retrieval irrespective of exercise (suggesting neural compensatory effects even at baseline), whereas, in the control group, such increase was only detectable after physical exercise. Critically, an exercise-related endocannabinoid increase correlated with task-related hippocampal activation in the control group only. In conclusion, healthy young individuals carrying the ε4 allele may present suboptimal associative memory performance (when compared with homozygote ε3 carriers), together with reduced plasticity (and functional over-compensation) within medial temporal structures.

## Introduction

The first hallmark of Alzheimer’s disease (AD) is memory loss. While spatial memory deficits are commonly reported (Lithfous et al. 2013; Parizkova et al. 2020), studies also highlight associative memory dysfunctions at the early stages of this degenerative disease (Sperling 2007). Late onset AD has a genetic component, with the APOE polymorphism currently corresponding to the most important genetic risk factor. This gene codes for apolipoprotein E, a molecule largely involved in the transport and metabolism of lipids in the body. Among the three isoforms of this polymorphism, ε4 is associated with an increased risk to develop late onset AD, whereas ε2 is protective against this disease, with ε3 being neither protective nor deleterious (Solomon et al. 2018). About 60% of the general population is ε3/ε3 homozygote, the ε4 allele is present in 15% to 20% of the worldwide population (Eisenberg et al. 2010), with strong geographical differences around the planet (with a mean of 6.8% in India to 22.1% in Ocenia; Singh et al. 2006). Carriers of this allele have a 3- to 12-fold increased risk to develop the disease, together with an earlier onset (mean age 68 as compared with 84 for non-carriers; Corder et al. 1993).

A recent study showed that young carriers of this allele (heterozygotes ε4/ε3) already presented impaired grid-cell-like representations, suggesting suboptimal entorhinal spatial coding despite intact behavioral performance (Kunz et al. 2015). Using another virtual navigation task, a follow-up study in ε4 carriers also found impairments in path integration, supposedly based on the head-direction system (Bierbrauer et al. 2020), involving hippocampus, entorhinal, but also posterior cingulate/retrosplenial cortices (Epstein et al. 2017; Mitchell et al. 2018). Other studies revealed that the grid-cell-like representations in the general population become degraded with older age, plausibly yielding path integration deficits (Stangl et al. 2018). Together, these observations point to a link between decreased grid-cell activity and age-related memory deficits, possibly preceding dementia. Yet, while asymptomatic young carriers of the APOE ε4 allele have impaired grid-cell-like representations, it is unclear whether and how memory performance in these individuals may be modified.

The medial temporal lobe is essential for not only spatial navigation, but also memory formation and recall (Cassilhas et al. 2016; Jeffery 2018). As mentioned above, it is also the first brain circuitry to be affected in AD. With the lack of effective treatment, the focus of some current research has been shifted to lifestyle factors that can prevent or delay the onset of the disease. Physical exercise is one of these factors which is easily accessible to the majority of the population and that has proven beneficial to neuronal and cognitive health throughout the lifespan (Smith et al. 2010; Ahlskog et al. 2011; Buchman et al. 2012; Roig et al. 2013). A longitudinal study in older adults documented that regular aerobic exercise (walking three times a week) can lead, after 12 months, to an increase in hippocampal gray matter volume, which itself was found to correlate with improved spatial memory (Erickson et al. 2011).

Several studies suggested that higher general fitness levels may attenuate cognitive deficits or risk for developing AD, particularly for the carriers of the APOE ε4 allele (Schuit et al. 2001; Rovio et al. 2005; Etnier et al. 2007; Prakash et al. 2015). Using brain imaging methods, the positive effects of physical fitness on semantic memory were found to correlate with increased glucose uptake, and to depend on APOE ε4 status, such that they were greater in ε4 carriers than in noncarriers (Smith et al. 2011; Deeny et al. 2012). Please note that while most of these studies were carried out on older volunteers—more than 55 years old, (Schuit et al. 2001; Etnier et al. 2007; Smith et al. 2011; Deeny et al. 2012; Woodard et al. 2012), recent data suggest that some deficits related to APOE ε4 carrier status can already be detected in young 18–30-year-old participants (Kunz et al. 2015; Bierbrauer et al. 2020).

Additionally, exercise-related benefits have been reported after acute physical exercise, with just one bout of physical exercise yielding enhanced memory performance and medial temporal lobe activity (van Dongen et al. 2016; Suwabe et al. 2017; Loprinzi 2019). Acute exercise also causes an increase in the release of different biomarkers including the endocannabinoid neurotransmitter anandamide (AEA), with higher anandamide levels after exercise correlating with increased hippocampal activation during an associative memory task (Marin Bosch et al. 2021) and better motor sequence memory (Marin Bosch et al. 2020).

Based on these diverse observations, we asked whether the behavioral and neural effects of acute physical exercise in young participants might be differentially modulated for individuals with an increased or low risk of developing AD later in life, i.e. their APOE status. To address this question, we designed a within-subject paradigm to evaluate the effects of a single session of exercise (compared with rest) on associative memory, brain activity, and blood biomarkers in healthy young heterozygotes ε3/ε4 (risk group) and homozygotes ε3/ε3 (control group) volunteers (matched for age, gender and BMI and fitness level). Each participant came to the lab twice, once for a rest and once for an exercise condition in counterbalanced order. We hypothesized (i) that the risk group would perform worse on the associative memory task, and (ii) that this difference could be linked to suboptimal medial temporal lobe activity in individuals of the genetic risk group, and/or to disrupted links with blood biomarkers (Marin Bosch et al. 2020, 2021). Additionally, we hypothesized (iii) that acute exercise would attenuate these group differences (if any).

## Materials and methods

### Participant recruitment

#### Initial recruitment

All participants gave written informed consent and received financial compensation for their participation, which was approved by the local ethics committee (Cantonal Ethics Committee of Geneva—CCER; project 14-240). Participants were all right-handed, non-smokers, free from psychiatric and neurological history, and had a normal or corrected-to-normal vision. All participants were within the normal ranges on self-assessed questionnaires for depression (BDI; Beck et al. 1961), anxiety (STAI; Spielberger 1983), and circadian typology (PSQI; Horne and Ostberg 1976).

#### Genetic prescreening

We took a saliva sample into an Oragene OG 500 tube from a total of 263 healthy volunteers who responded to our flyer and who were between 18 and 35 years old, had a BMI between 18 and 25, did not have a history of psychiatric or neurological disorder, were not claustrophobic, and did not have a fear of needles. We did a qPCR to discriminate between the isoforms of APOE with the primers rs429735 and rs7412.

#### Final recruitment

To determine our required sample size, we performed a power analysis (G*Power 3.1.9.7) to compare two matched groups differing only by genotype. We expected that the performance of the risk group should be below that of the control group therefore we used a one-tailed test which yielded a sample size of 45 participants (with standard effect size of 0.5, α = 0.05 and β = 0.05), with a more conservative two-tailed test, this yields a total sample size of 54 participants. This is what we aimed for, and our final sample comprises 54 participants, corresponding to the requirement of the two-tailed test.

Out of the prescreened participants, we included 26 participants with the ε3/ε4 genotype (risk group). We also selected 28 participants with the ε3/ε3 genotype (control group), matched with the risk group for age, gender and BMI and fitness level (see Table 1 for characteristics of all 54 participants). All participants were non-smokers, right-handed, without any history of neurological or psychiatric disorders. Participants were informed that we preselected them based on some genetic polymorphisms but were not aware of the specific polymorphism targeted. All participant genotyping, selection, matching, and testing was done in a double-blind manner. Neither the experimenters nor the participants knew the group the latter belonged to throughout the protocol (from data acquisition to final analyses).

**Table 1:** Individual data of all participants.

**Subject (as per order)**	**Genotype**	**Gender**	**Age (years-old)**	**BMI**	**Fitness (maximal power output during VO2max normalized to 100 by gender)**
**1**	ε4/ε3	f	20	19.69	50
**2**	ε3/ε3	f	22	21.36	62
**3**	ε4/ε3	f	22	19.54	48
**4**	ε3/ε3	f	20	19.08	36
**5**	ε4/ε3	f	22	21.32	48
**6**	ε3/ε3	f	24	20.76	57.2
**7**	ε4/ε3	f	20	19.27	57.2
**8**	ε3/ε3	m	23	22.73	72.86
**9**	ε4/ε3	f	35	22.31	68
**10**	ε3/ε3	m	23	24.38	64.29
**11**	ε4/ε3	f	22	18.98	64
**12**	ε4/ε3	m	36	25.26	48.57
**13**	ε3/ε3	f	22	21.80	44
**14**	ε3/ε3	f	20	21.67	66
**15**	ε3/ε3	f	22	21.22	52
**16**	ε4/ε3	m	22	23.45	54.29
**17**	ε4/ε3	f	19	19.95	60
**18**	ε3/ε3	m	22	20.06	68.57
**19**	ε4/ε3	m	22	22.59	77.14
**20**	ε4/ε3	f	21	20.45	64
**21**	ε3/ε3	f	23	19.84	68
**22**	ε3/ε3	m	22	23.39	60.86
**23**	ε4/ε3	m	21	20.16	64.29
**24**	ε4/ε3	f	22	20.20	50
**25**	ε4/ε3	m	22	24.69	68.57
**26**	ε4/ε3	m	23	23.84	71.43
**27**	ε3/ε3	f	23	18.75	42
**28**	ε3/ε3	m	23	24.93	65.71
**29**	ε3/ε3	f	20	19.20	48
**30**	ε4/ε3	m	25	23.04	90
**31**	ε4/ε3	f	20	23.51	90
**32**	ε3/ε3	m	20	20.75	47.14
**33**	ε3/ε3	m	21	23.45	59.14
**34**	ε4/ε3	m	22	19.38	40
**35**	ε3/ε3	f	19	22.76	44
**36**	ε3/ε3	f	20	19.13	68
**37**	ε3/ε3	f	20	22.43	59.2
**38**	ε4/ε3	m	27	21.89	51.43
**39**	ε3/ε3	f	26	24.03	44
**40**	ε3/ε3	m	21	21.86	60.57
**41**	ε3/ε3	m	21	21.39	61.43
**42**	ε3/ε3	f	21	21.34	48
**43**	ε3/ε3	f	19	21.77	54
**44**	ε3/ε3	f	24	21.26	44
**45**	ε4/ε3	f	30	21.41	60
**46**	ε3/ε3	f	22	20.31	40
**47**	ε3/ε3	f	20	23.01	64
**48**	ε4/ε3	f	23	24.34	84
**49**	ε4/ε3	m	30	23.71	62.86
**50**	ε4/ε3	f	21	22.63	76
**51**	ε3/ε3	f	23	24.34	78
**52**	ε4/ε3	m	18	21.45	48.57
**53**	ε4/ε3	f	20	19.83	76
**54**	ε4/ε3	f	20	18.05	58.8

### Experimental protocol

**Fig. 1:**
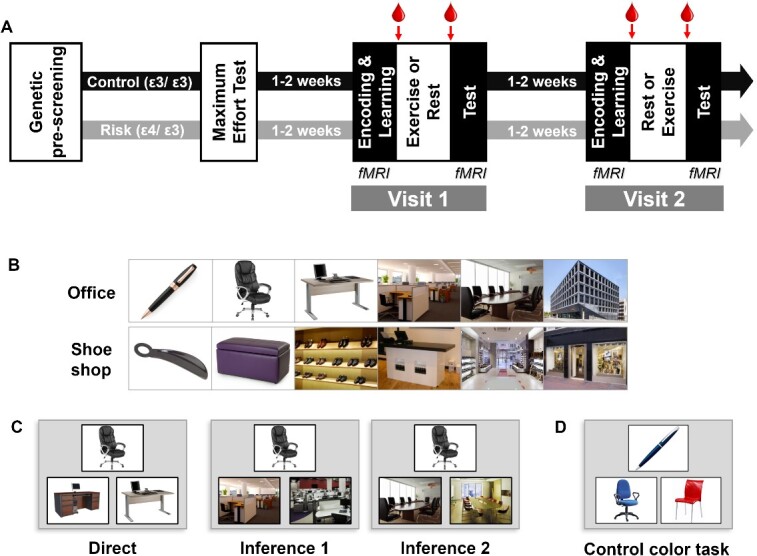
Experimental procedure. (A) Timeline of the experiment. Arrows indicate times for blood samples., (B) Two examples of sequences of images in the associative memory task. (C) Examples for the different types of trials of the test part, with increasing relational distance in the sequence from left to right. Participants had to choose between the two bottom pictures, the one belonging to the same learned sequence as the top one. (D) One example of a control color task where participants had to choose between the two bottom pictures, the one of the same color as the top one.

The full protocol once participants were selected was composed of three visits: one introductory and two experimental visits (see Fig. 1A).

#### Introductory visit

The introductory visit comprised a maximum effort test on a cycle ergometer and a habituation to our cognitive tasks to minimize learning effects. The maximum effort test was used to determine each participant’s individual maximum heart rate (FcMax), corresponding to their individual fitness level, and to individually tailor the workload imposed during the exercising part. We aimed at a workload of 60% FcMax for every participant, corresponding to a moderate intensity of physical effort. The maximal effort test was performed on a cycle ergometer (TechnoGym exc 700i, TechnoGym, Cesena, Italy), while continuously monitoring heart rate by cardiotachography (Polar RS 800 CX, Polar, Finland) and electrocardiogram (Biopac Systems, Inc.). Blood pressure was also continuously monitored using a multiparameter patient monitor (Cardioline vsign200, Cardioline SpA, Milano, Italy). The test started with a 10-min warm-up at 30 W, after which the resistance was increased by 10 W every minute until exhaustion.

#### Experimental visits

For both experimental visits, participants came in at 8 AM on an empty stomach and had a standardized breakfast which consisted of black coffee or tea, white bread, and jam (as in Marin Bosch et al. 2021). The amount of each item was not limited, but they were instructed to take the same amount at both visits. They were placed into the MRI scanner at 9 AM, and first performed an encoding session during which they saw all 8 series of 6 pictures once (Fig. 1B). The encoding session was immediately followed by a 2-alternative forced choice (2AFC) learning session with feedback as described previously (Igloi et al. 2015; Bosch et al. 2017; Marin Bosch et al. 2021). FMRI was acquired during encoding and learning sessions. Participants were then put out of the scanner and, at 9:50 AM, a qualified nurse took a first blood sample. At 10:00 AM participants were equipped with a Polar RS800CX N device to measure heart rate and asked to rest or exercise. For moderate intensity exercise, each participant pedaled for 30 min at a cardiac frequency of 60–65% of their FcMax. For the rest condition, participants sat on a chair and were allowed to quietly look at magazines for 30 min. To assess subjective levels of effort, we asked our participants after 15 min in each Exercising Condition (exercise and rest) to rate their perceived effort on a scale of 0–10 (0 being no effort and 10 being the most strenuous effort they could imagine). At 10:45 AM (i.e. 15 min after the exercise or rest period ended), the qualified nurse took a second blood sample. At 12:00 PM, participants underwent a second fMRI session during which memory for the associative task was tested using a 2AFC task on 72 pairs of pictures with different relational distances (direct, inference of order 1 and order 2; 24 trials of each category, Fig. 1C). Sixteen control color-based trials were also included, in which the depicted elements were of a given color (red, blue, or green) and participants had to choose among two pictures which one was of the same color as the target picture (Fig. 1D, control color task).

### Analysis

#### Behavior

Analysis of the associative memory performance was conducted similarly to Marin Bosch et al. (2021). In short, we first calculated performance during the learning phase and during the test phase as percentage of correct responses per inference level. Then, to account for potential initial differences of performance during learning, we computed a normalized score of performance, by dividing performance at test by performance at learning. We ran repeated-measures ANOVAs on this score with Exercising Condition (exercise/rest) and Inference (direct/inference1/inference2) as within-subject factors and Genotype (risk/control) as between-subject factor and Fitness (normalized by gender as continuous predictor). All analyses were performed using Statistica (Version 12, www.statsoft.com, StatSoft, Inc. TULSA, OK, USA).

#### Biomarkers

Before and after each exercise or rest condition, 2.5 mL of blood were collected into a BD Vacutainer K_2_EDTA 5.4 mg tube. This tube was immediately centrifuged for 10 min at 8009 g at 4°C, the supernatant (plasma) was taken in aliquots of 200 μL. All samples were then frozen and stored at −80°C until analysis. The endocannabinoid AEA levels were determined from 100 μL of plasma by liquid–liquid extraction. This was followed by liquid chromatography (Ultimate 3000RS, Dionex, CA, USA) and mass spectrometry using a 5500 QTrap® triple quadrupole/linear ion trap (QqQLIT) mass spectrometer equipped with a TurboIon-SprayTM interface (AB Sciex, Concord, ON, Canada) as described previously (Thomas et al. 2009; Quercioli et al. 2013).

#### Imaging acquisition

We acquired simultaneous multi-slice echo-planar images with a TR of 1900 ms on a 3 T Magnetom TIM Trio Scanner (Siemens, Erlangen, Germany), voxel size 2 × 2 × 2 mm^3^. We also collected a high-resolution T1-weighted anatomical image (TR/TI/TE/Flip angle = 1900 ms/900 ms/2.32 ms/9°, FOV = 230 mm, resolution = 256 × 256, slice thickness = 0.9 mm, 192 sagittal slices).

#### Functional MRI analysis

All fMRI scans were preprocessed using standard procedures of realignment, co-registration, slice timing, normalization and smoothing using SPM12 (Wellcome Department of Imaging Neuroscience, London, UK), similarly to Marin Bosch et al. (2020). A general linear model (GLM) approach was then used to compare conditions of interest at the individual level. Each individual GLM included correct trials separated according to Relational Distance (direct, inference 1, inference 2), control trials, and incorrect trials (pooled across Relational distance). Additionally, six movement regressors, five heart rate regressors, and one breathing regressor (to correct for potential heart- and breathing-related artifacts; Glover et al. 2000; Birn et al. 2008) together with Fitness (normalized by gender as continuous predictor) were added as regressors of non-interest. Then, contrasts between conditions of interest from each participant were entered into a second-level random-effects analysis. We report activations at 0.001 uncorrected, with a minimal cluster size of 10 contiguous voxels, that survive subsequent small volume correction using a bilateral hippocampal mask from JuBrain Anatomy Toolbox (fz-juelich.de/en/inm/inm-7/resources/jubrain-anatomy-toolbox, Julich, Germany).

## Results

### Behavior

**Fig. 2:**
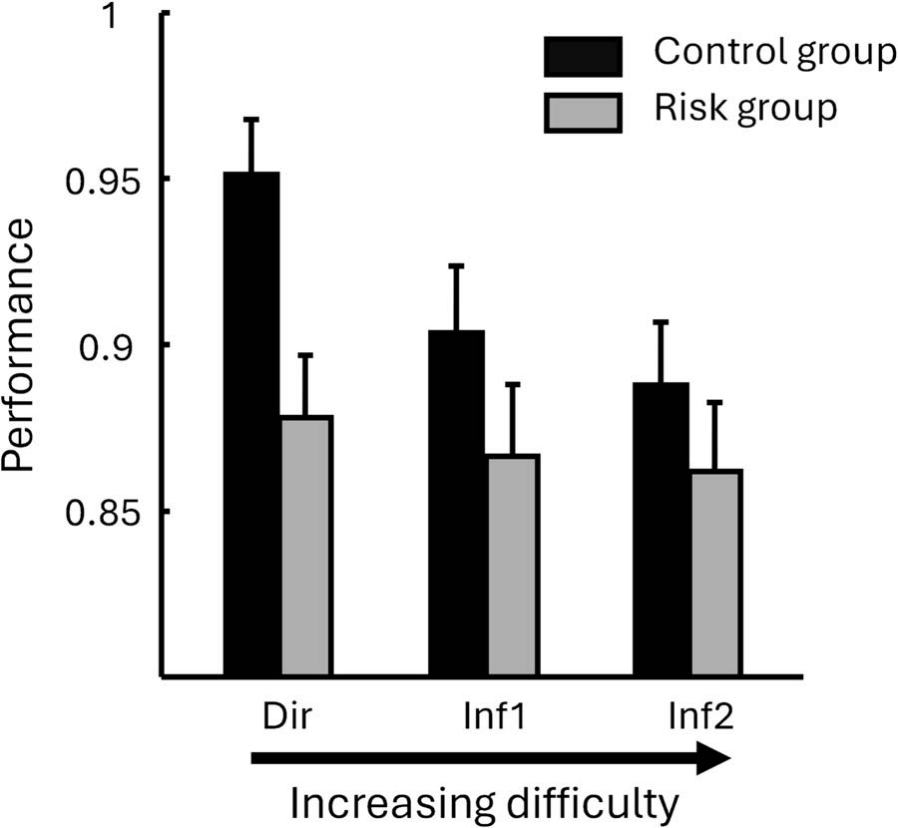
Behavioral results at test for the risk (ε3/ε4) and control (ε3/ε3) groups, and for stimuli of increasing relational distance from the left to right. The control group performed significantly better than the risk group. Note that because there was no effect of exercising (condition exercise, rest) nor any interaction with exercising condition, the data from both exercise and rest were pooled.

We conducted a within-subject ANOVA on normalized performance scores for the associative memory test, with Exercising Condition (exercise/rest) and Relational Distance (direct/inference 1/inference 2) as within-subject factors, Genotype (risk/control) as between-subject factor and Fitness (maximal power output during VO2max normalized to 100 by gender, see Table 1) as continuous factor. We report no effect of Exercising Condition (*F*(1, 51) = 0.03, *P* = 0.863), no effect of Relational Distance (*F*(2, 102) = 0.706, *P* = 0.496), but a main effect of Genotype (*F*(1, 51) = 5.374, *P* = 0.024) with control participants performing better than risk participants (Fig. 2). None of the interactions yielded significant differences all *P* > 0.1.

#### Biomarkers

An ANOVA with Exercising Condition (exercise/rest) as within and Genotype (ε3/ε4) as between subject factors was performed on the difference between the second (after exercise or rest) and the first baseline sample for AEA blood levels. This analysis revealed a significant effect of Exercising Condition (*F*(1, 52) = 19.31, *P* < 0.001), no effect of Genotype (*F*(1, 52) = 0.27, *P* = 0.61), and no interaction (*F*(1, 52) < 0.001, *P* = 0.99; Fig. 4A).

**Fig. 3:**
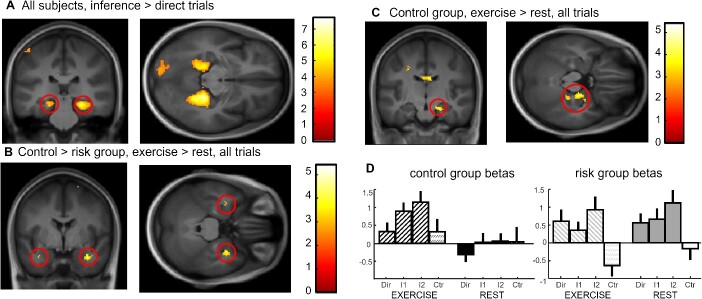
Imaging results at test. (A) Inference 2 and inference 1 trials versus direct trials for both groups and both exercising conditions showing bilateral parahippocampal and hippocampal activation. (B) Interaction control vs risk for exercise vs. rest conditions across all associative memory trials showing bilateral medial temporal lobe activation. (C) Effect of exercise versus rest across all associative memory trials for the control group showing right hippocampal activation at 20, −16, −20. (D) Beta values for the hippocampal activation in (C) for each trial type and Exercising Condition separately showing a clear difference between conditions in the control group but a similarly high hippocampal activity in both exercise and rest for the risk group.

**Fig. 4:**
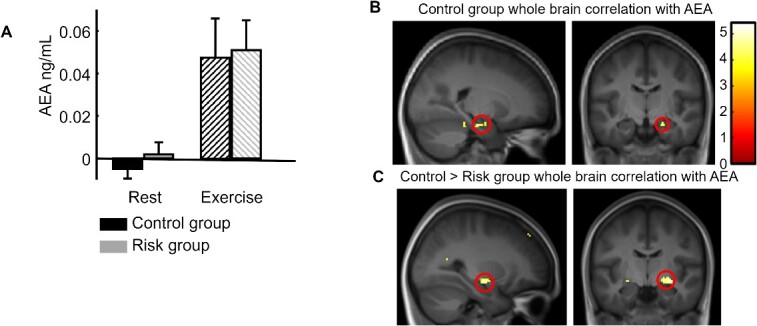
Endocannabinoid results. (A) Increase in the AEA plasma concentration after exercise compared with after rest for both groups. (B) Whole-brain correlation for the control group between individual AEA increases (after exercise minus after rest) and the fMRI contrast between exercise and rest for all associative memory trials (direct inference 1 and inference 2), showing a significant correlation in the right hippocampus (replication of Marin Bosch et al. 2021). (C) Group difference (control > risk) for whole-brain correlations with AEA, showing the implication of the right medial temporal cortex (at the superior limit of the hippocampal formation).

#### Functional MRI

To confirm the contribution of para/hippocampal regions to associative memory, we first computed the effects of Relational Distance over all subjects by contrasting inference 1 and inference 2 trials versus direct trials ([Inf1 + Inf2]-2xDirect). We report an activation of the bilateral parahippocampus, extending into the hippocampus (Fig. 3A, Table 2 for all activations). Next, to test for any differential effect of physical exercise on risk and control groups, we compared activation after exercise versus rest for control versus risk group irrespective of Relational Distance (direct, inference 1, inference 2 trials together) and found a bilateral activation of the medial temporal lobe (the cluster of activation is in the hippocampus, partially extending into the amygdala, with the main statistical peak at 32*x*, −2*y*, −26*z* located in the amygdala; see Fig. 3B, SVC-corrected). The reverse activation yielded no significant voxel. To further understand the patterns of activation underlying the interaction results, we computed the same contrast (exercise vs rest) within each group separately. While the control group showed a significant right hippocampal activation (20*x*, −16*y*, −20*z* SVC-corrected; see Fig. 3C), there was no significant medial temporal activation within the risk group. Please note there is also a small activation in the amygdala for this contrast (at 32*x*, −2*y*, −28*z*), but which is smaller than the minimal cluster size (7 voxels). This suggests that exercise did not modulate hippocampal recruitment during the associative task in the risk group. To illustrate the differences between the groups, we display the beta values at the hippocampal peak (20*x*, −16*y*, −20*z*) in Fig. 3(D), for all Groups, Exercising Conditions and Relational Distances (see Table 2). The pattern of the beta values suggests that the absence of hippocampal modulation by exercise in the risk group might be related to an augmented (potentially compensatory; see Discussion) involvement of the hippocampus after both exercise and rest.

**Table 2:** fMRI activations for the main contrasts of interest.

**Brain region**	**Lat.**	**Cluster size**	**Unc. *P*-value**	**SVC *P*-value**	**Peak T**	**Peak Z**	**X**	**Y**	**Z**
Increasing Relational Distance ([inf. 1 + inf. 2] > [2*direct]) for both Genetic Groups and Exercising Conditions— Fig. 3(A)									
Precuneus	Right	799	<0.001		9.11	7.03	18	−52	14
Lingual gyrus	Left	576	<0.001		8.36	6.65	−24	−46	−8
**Hippocampus**	Left		<0.001	0.005	7.89	6.39	−18	−34	−12
**Hippocampus**	Right	1079	<0.001	<0.001	8.33	6.63	28	−44	−4
Parahippocampus	Right		<0.001		7.97	6.43	24	−36	−10
Calcarine gyrus	Left	495	<0.001		7.13	5.94	−16	−54	12
Occipital gyrus	Left	270	<0.001		4.80	4.36	−12	−86	−2
**([control > risk] > [exercise > rest]) for all Relational Distance trials—** Fig. 3(B)									
Thalamus	Right	72	<0.001		4.99	4.49	10	−22	22
Left cerebellum IV–V	Left	27	<0.001		4.01	3.72	−6	−44	−12
**Medial temporal lobe**	Right	11	<0.001		3.54	3.33	32	−2	−26
**[exercise > rest] in the control group for all Relational Distance trials—** Fig. 3C									
Parahippocampus	Left	18	<0.001		4.20	3.88	−30	−40	−4
**Hippocampus**	Right	17	<0.001	0.024	3.82	3.57	20	−16	−20
Angular gyrus	Right	44	<0.001		3.72	3.48	50	−64	32
Superior medial prefrontal gyrus	Right	39	<0.001		3.69	3.46	4	52	2
**[exercise > rest] in the risk group for all Relational Distance trials**									
Middle frontal gyrus	Right	36	<0.001		4.39	4.033757	42	30	44
**Whole-brain correlation between AEA changes and contrast [exercise > rest] in the control group for all Relational Distance trials—** Fig. 4									
**Hippocampus**	Right	13	<0.001	0.026	5.11	3.72	8	−14	−20

In our previous work (Marin Bosch et al. 2021), we found that concentration changes in AEA after exercising correlated with increase in hippocampal activation after exercise. Here we applied a similar whole brain correlation approach using individual AEA concentration changes, separately for the risk and the control group. For the risk group, not a single voxel correlated with AEA changes whereas, for the control group, a significant positive correlation was observed in the right hippocampus (Fig. 4B, Table 2, SVC-corrected). Please note that, here as well, there was another small peak in the amygdala (32*x*, −3*y*, −25*z*), but which did not survive small volume correction. Additionally, a statistical contrast between these group results (i.e. comparison of correlation slopes) confirmed the implication of the right medial temporal cortex (at the superior limit of the hippocampal formation, Fig. 4C).

## Discussion

In the present study, we used an associative memory task in fMRI to assess the effects of a single session of exercise on two groups of young individuals at either high genetic risk (carrying one copy of the APOE ε4 allele) or control (APOE ε3 homozygotes) of developing AD in later life. One main strength of the present study design is that it involved a careful pre-screening of participants so as to compare two groups of individuals matched for gender, fitness, BMI, and age, but differing regarding their APOE polymorphism (risk group: APOE ε3/ε4 heterozygotes vs control group: APOE ε3 homozygotes). Moreover, unlike cross-sectional designs, here the effects of physical exercise on memory, fMRI activation, and AEA concentration changes were assessed within each individual (within-subject design), before the results from the matched groups were compared. Additionally, to limit variability within the risk group, we did not include ε4/ε4 homozygotes because their risk for developing AD is even greater than that of ε3/ε4 individuals (i.e. the impact of this extra ε4 copy is substantial) and because they represent less than 1% of the population. Similarly, the control group was strictly composed of ε3 homozygotes.

Confirming our initial hypothesis for behavior (see Introduction section), the risk group had poorer associative memory performance compared with control group. At the brain level, the interaction between genetic group and exercising condition revealed that, during the associative memory task, the control group showed an increased hippocampal (mostly right) activation after exercise (compared with rest), whereas hippocampal activity was elevated after both exercise and rest in the risk group. This result confirms our initial hypothesis pertaining to the differential modulation of fMRI activity across the groups, and partially our hypothesis that exercise would attenuate differences between the groups. Indeed, although fMRI does not provide absolute measures of brain activity, the fMRI results seem to indicate that exercise would tend to somewhat “normalize” brain activity in the risk group. Specifically, the latter displayed elevated hippocampal activation (for the associative memory trials as compared with the no-memory, control trials), irrespective of the exercising condition, whereas the control group showed increased activation selectively after exercise (as we previously reported for participants without genetic characterization, among whom only about 1/5 may plausibly have been ε4 carriers (Eisenberg et al. 2010)). Further, while we replicate previous findings pertaining to hippocampal activation correlating with AEA levels in control individuals, no such correlation was present in the risk group. Taken together, the present results evidence worse memory performance with concomitant suboptimal medial temporal lobe recruitment in a group of individuals with a high genetic risk of developing late AD.

### Memory function in young APOE ε4 carriers

We report poorer associative memory performance in young individuals carrying an APOE ε4 allele, which is believed to increase the likelihood of developing AD later in life, as compared with young APOE ε3 homozygotes. Different studies indicated that the APOE ε4 allele may have a form of compensatory pleiotropy at young age with better overall cognition, especially attentional capacities (Rusted and Carare 2015), speed of processing (Marchant et al. 2010), mental arithmetic (Puttonen et al. 2003), verbal fluency (Alexander et al. 2007; Marioni et al. 2016), and decision making (Marchant et al. 2010). Cacciaglia et al. (2023) showed decreased connectivity specifically in the left medial temporal lobe network and centered on the hippocampus proper correlating with the higher number of ε4 alleles in the genome of the participants (zero ε4 allele for ε3/ε3 participants, one ε4 allele for ε3/ε4 and ε2/ε4 participants, and two ε4 alleles for ε4/ε4 participants), but this report also pertains to older participants (mean age above 50 years old). Yet, while late onset AD is primarily characterized by loss of hippocampal cells and memory impairments (Niikura et al. 2006), only few well-powered and controlled experimental studies recently suggested suboptimal memory capacities (and hippocampal activation patterns) in APOE ε4 carriers at a young age (Kunz et al. 2015; Bierbrauer et al. 2020); note however that the reported memory deficits appeared to be very subtle. The rarity of such studies may relate to the general belief that memory deficits in these individuals may only become measurable at older age (Smith et al. 2011; Solomon et al. 2018; Stangl et al. 2018). Hence, the present study adds important evidence supporting altered declarative memory function in individuals at genetic risk for AD. Furthermore, since our task loaded most selectively on memory, with no or minimal demands regarding speed of processing or selective attention, it may have impeded potential behavioral compensatory mechanisms and thus allowed the expression of memory deficits in young APOE ε4 carriers. Concerning the impact of genetic risk on memory at older age, the findings appear disparate, with some studies suggesting a beneficial effect of the ε4 allele on episodic (Mondadori et al. 2007) and prospective memory (Evans et al. 2013) at young age. Whereas others like Li et al. (2019), found rather lower cognitive performance for APOE ε4 carriers, this was specific to participants from lower educational levels.

### No beneficial effects of physical exercise on associative memory in young APOE ε4 carriers

Physical exercise is well known to have overarching positive effects on cognition throughout the lifetime; see Singh et al. (2019) for results in children, Prakash et al. (2015) during adult lifetime, and Dauwan et al. (2021) in healthy aging and chronic illnesses. Importantly it is also recognized that genetic components may modulate these effects (Prakash et al. 2015). As mentioned in Introduction, not only long-term but also acute physical exercise has recently been shown to be associated with memory and hippocampal function benefits for normal healthy volunteers (van Dongen et al. 2016; Suwabe et al. 2017; Marin Bosch et al. 2020, 2021), and in a recent integrated model by Loprinzi (2019).

Generally previous studies linking APOE status and physical exercise have focused on a population of healthy older volunteers and use fitness levels as cross-sectional variables (Schuit et al. 2001; Etnier et al. 2007; Smith et al. 2011; Deeny et al. 2012; Woodard et al. 2012), therefore not looking at the effects of a physical exercise intervention, as we do here. Yet, because some cognitive deficits related to APOE ε4 carrier status can already be noticeable at younger age (see previous section and Kunz et al. 2015; Bierbrauer et al. 2020), we aimed to assess whether a single session of exercise might differentially affect memory and brain plasticity (particularly in the hippocampus) in risk and control groups.

Unlike our previous study (Marin Bosch et al. 2021), here we did not see any main effect of physical exercise on associative memory performance. One main difference between the present and our previous study is that in the latter all participants were well trained, whereas in the present study, participants had variable general fitness, including many totally sedentary participants. Hence, to ensure that all participants of the present study could complete the whole experimental protocol, we slightly reduced the targeted exercising intensity from 65% of VO2max (in our previous study) to 60% of VO2max, which was still perceived as moderate intensity exercise (exercise session, mean+/-STD: 4.3+/−1.4; rest session: 0.2+/−0.6). With participants mainly sedentary and some of them pedaling at the minimal intensity (i.e. 30 W corresponding to almost no resistance) to stay in the correct heart rate range, we may speculate that the physiological stress induced by the 30-min exercise may have been more important in this study than in our previous one. Our recruitment was performed so as to match risk and control groups for fitness levels and gender, further, to ensure that our effects are not driven by different fitness levels, all our behavioral and fMRI analyses include participant’s fitness as cofactor.

While exercise did not seem to affect behavior in either group included in the present study, the fMRI results revealed an interaction effect in the hippocampus between exercising condition and genotype, suggesting that the risk group did not show increased hippocampal activity after exercise as compared with after rest, as was found in the control group. However, the hippocampal beta values across trial types and exercising conditions may rather indicate that the risk group activated the hippocampus more during both exercising conditions, the interaction being due to higher activation for the risk group during rest. Converging with data from another fMRI study in the same genetic population (Kunz et al. 2015), one plausible interpretation of the present pattern of results is that elevated hippocampal activity in the risk group might compensate for suboptimal memory, even at baseline rest. This is also consistent with Caccaglia et al. (2023) suggesting network-based functional compensation mainly in the temporal lobe in APOE ε4 carriers. Further, by combining these imaging results with AEA biomarker measures, we found different relationships as a function of genetic group. While hippocampal activation increase correlated with exercise-related AEA increase in the control group (replicating our previous findings on non-genetically characterized, young healthy participants; Marin Bosch et al. 2021), in the risk group, AEA increases was not proportional to hippocampal activity. AEA signaling is known to modulate plasticity mechanisms in the central nervous system (Chevaleyre et al. 2006), particularly in the hippocampus (Chevaleyre and Castillo 2004; Katona and Freund 2012; Piette et al. 2020). Thus, our present findings confirm previous proposals that AEA increase after exercise relates to hippocampal-plasticity and memory performance (Ferreira-Vieira et al. 2014; Marin Bosch et al. 2020, 2021), and suggest that these interactions may be more consistent or less effective in APOE ε4 carriers already at a young age. Thus, high activation of the hippocampus after both rest and exercise in participants at genetic risk, i.e. APOE ε4 heterozygotes may reflect a neural compensation for suboptimal memory function.

### Implication of the amygdala

While we mainly discussed how hippocampal activity was modulated during the memory task as a function of genotype and exercise, the effects of these factors sometimes also encompassed the neighboring amygdala. Regarding the influence of APOE polymorphism on amygdala function, a couple of studies suggested that APOE e4 carriers may have decreased gray matter density in the amygdala (Honea et al. 2009; Tang et al. 2015), but note that these effects have been reported in older participants (over 60 years old). Regarding the effects of physical exercise on amygdala responses during the memory task, it is known that moderate intensity exercise (as was used in the present study) increases endocannabinoid signaling (Marin Bosch et al. 2020, 2021), which may also implicate the amygdala. If in our rather sedentary sample, the exercising session has indeed had a stressful effect this may be related to endocannabinoids-related plasticity mechanisms that have been reported in rodents in both the hippocampus and the basolateral amygdala, in relation to fear response (Segev et al. 2018; and see Gunduz-Cinar 2021 for a review).

### Limitations

The present experiment had several limitations. First, we included participants with various fitness levels and of both genders, thus yielding a more heterogeneous sample than our previous studies, where we included regular exercising participants. We corrected for potential effects of this factor by matching both the risk and control groups for fitness levels and by including the individual fitness level as a cofactor in all analyses. Second, while we waited for 45 min at the end of the physical exercise or rest session before bringing our participants back into the scanner, we cannot rule out that some of our participants were still experiencing some lingering effects of the exercise task. However, subjective exertion ratings performed in the middle of the exercise and rest periods indicated (i) no difference between the groups and (ii) moderate exertion during exercise (at 4 on a scale from 0 to 10). Third, we only recruited participants with two specific genotypes (ε3/ε3 and ε4/ε3), which would in principle account for about 80% of the population (Eisenberg et al. 2010) and represented 84.41% of the 263 participants that we genotyped. As the proportion of ε2 allele carriers in the population is lower (about 10–15%, 13.63% in our initial sample, comprising mainly ε2/ε3 genotypes but a few ε2/ε2 and ε2/ε4 genotypes as well) and homozygotes ε4/ε4 genotypes comprise less than 1% of the population (0.38% in our initial sample), including these other genotypes would have considerably complicated the recruitment, especially for such a complex design where all participants needed to come to the laboratory at least 3 times with associated increased dropout risk. Hence, the findings from the comparison between the two most frequent APOE genotypes, as was done here, cannot be generalized to other APOE genotypes, or to other genetic polymorphisms, which may also play a role in the effects of exercise on memory, such as BDNF Val66Met for example (Erickson et al. 2013).

Note finally that data acquisition was done during the Covid period, which did not only complicate the experimental process, but also led to increased drop-out rate and delays in data acquisition due to lockdown, sanitary restrictions or contact tracing hazards of the experimenters or the volunteers (at least five participants so about 10% of our sample had to be discontinued during the 2020–2021 lockdowns). Despite this unfortunate and constraining situation, we managed to include the data from 54 participants, forming two carefully matched genetically characterized groups, who completed the experiment in its entirety. While this sample size may be considered low for genetic studies, here our main aim was to use brain imaging to assess neural responses during a memory task in two carefully matched groups that differed for one main variable, i.e. their APOE polymorphism in a within-subject paradigm comparing exercise to rest. The experimental approach that we used is reliable and valid for any neuroimaging study comparing 2 groups that differ on an individual dimension that is considered to potentially influence a particular cognitive ability or behavior. We therefore had strong a priori hypotheses on differences in medial temporal brain activity between our two groups, which we tested here.

## Conclusion

In conclusion, our findings highlight that healthy young individuals with a single copy of the ε4 allele exhibit poorer performance on associative memory tasks compared with control participants. Additionally, we observed distinct patterns of hippocampal activity, which may suggest compensatory mechanisms at work in these individuals who are genetically predisposed to AD. Future research endeavors should delve into characterizing these mechanisms and their implications for individuals at genetic risk for AD, shedding light on the role of physical activity in shaping cognitive health over time.

## Data Availability

Data supporting this study is available from the corresponding author upon reasonable request.
